# A 14-3-3 Protein-Encoding Gene, *BdGF14g*, Confers Better Drought Tolerance by Regulating ABA Biosynthesis and Signaling

**DOI:** 10.3390/plants12233975

**Published:** 2023-11-26

**Authors:** Yang Zhang, Yuan He, Hongyan Zhao, Yan Zhang, Jing Yang, Xingqi Ou, Jinlong Zhang, Qidi Zhu

**Affiliations:** 1 School of Agriculture, Henan Institute of Science and Technology, Xinxiang 453003, China; tstcedufeiyang@163.com (Y.Z.); ouyangxq@163.com (X.O.); 2 The Genetic Engineering International Cooperation Base of Chinese Ministry of Science and Technology, The Key Laboratory of Molecular Biophysics of Chinese Ministry of Education, College of Life Science and Technology, Huazhong University of Science & Technology, Wuhan 430074, China; circlecirclehe@163.com (Y.H.); zhaohy@hust.edu.cn (H.Z.); 3 School of Life Sciences, Henan Institute of Science and Technology, Xinxiang 453003, China; yuansuqixiang@163.com (Y.Z.); yangjinghist@163.com (J.Y.)

**Keywords:** BdGF14g, abscisic acid, drought stress, ROS-scavenging system

## Abstract

Abscisic acid (ABA), a phytohormone, enacts a cardinal function in coping with abiotic stress. 14-3-3 proteins can interact with ABA-responsive-element-binding transcription factors (ABFs), a chief constituent of ABA signaling, and play critical roles in the dehydration response involving ABA signaling. Meanwhile, whether and how 14-3-3 proteins regulate ABA signaling to respond to aridity stress is yet to be fully investigated. Herein, *BdGF14g*, a 14-3-3 gene induced by ABA, H_2_O_2_, and PEG treatments, was identified in *Brachypodium distachyon* (*B. distachyon*). Overexpression of *BdGF14g* improved drought stress tolerance in tobacco plants, with a higher survival rate, longer root length, enhanced cell membrane stability, and increased antioxidase activity compared with non-transgenic controls in coping with dehydration. Both drought and exogenous ABA treatments resulted in smaller stomatal apertures in *BdGF14g*-transgenic lines. Additionally, when an ABA biosynthesis inhibitor was added, the better growth statuses, less H_2_O_2_ accumulation, and higher activities of catalase and superoxide dismutase under mannitol stress disappeared. Moreover, BdGF14g interacted with NtABF2, upregulated the endogenous ABA content, and enhanced the transcription of ABA-related genes, including *NtNCED1*, a crucial ABA biosynthesis gene, under drought conditions. In conclusion, BdGF14g acts as a positive factor in the water deficiency response by affecting ABA biosynthesis and signaling in tobacco plants.

## 1. Introduction

Harsh environments, including drought, restrict plant growth and development, especially for cultivating staple crops, and cause agricultural yield loss. Plants use a series of signal transduction networks to cope with various stress conditions. Recent research has reported that a group of adaptor proteins, 14-3-3s, play crucial roles in responses to stresses. According to the numbers of introns and exons, the 14-3-3 family genes are divided into non-ε and ε types [1]. The 14-3-3 family proteins usually form homologous or heterologous dimers to exhibit their functions, and some plant crystal structures that are similar to those in animals have been obtained [2,3,4]. Additionally, 14-3-3 proteins are highly conserved in different plant species and within the same species of plants, and their interactions with respective phosphorylated forms lead to the modulation of their activities and stabilities, the localization of targets, the substrate specificity of themselves or their derivatives, and the complex formation or disassembly of macromolecules [5,6].

Recent studies have shown that 14-3-3 proteins are involved in light signal transduction, plant hormone regulation, and plant growth and development. The activity of activated H^+^-ATPase [7] and nitrate reductase can be inhibited by 14-3-3 proteins [8,9]. In pepper (*Capsicum annuum*), 14-3-3 proteins interact with CaWRKY58 and activate CaWRKY58 transcription to cope with low-phosphorus (LP) stress [10]. Similarly, under LP conditions, OsGF14b responds to qPE9-1, exhibiting a higher phosphorus uptake by modulating root elongation [11]. Moreover, 14-3-3 proteins such as florigen Hd3a, NPH3, and phyB-PIF3 receptors are involved in light signal transmission [12,13,14]. Under long-day conditions, these proteins form a ternary florigen activation complex (FAC) with OsFD1 and RFT1 to promote flowering [15]. In *Arabidopsis*, 14-3-3η participates in the programmed cell death induced by H_2_O_2_ treatment [16], whereas 14-3-3k/j modulates autophagy dynamics by facilitating ATG13a and ATG13b degradation [17]. Under biotic stress, besides the expression of *GF14f*, the expression of *GF14e*, *GF14b*, and *GF14c* is induced by *Pyricularia oryzae* and *Xanthomonas oryzae* infection [18]. OsGF14f confers tolerance to the leaf blast and bacterial blight, and the interaction of OsGF14s with XopX or XopQ is necessary for the suppression of rice innate immunity during *X. oryzae pv. oryzae infection* [19,20].

Several studies have shown the vital role of 14-3-3 proteins in coping with abiotic stresses including salt and drought stress. In *Arabidopsis*, 14-3-3λ/κ functions under salt conditions via interacting with SOS2 [21]. The heterologous expression of *At14-3-3λ* in cotton imparts more drought stress tolerance to transgenic cotton than that of wild-type (WT) cotton [22]. The increased tolerance of *GRF9, ZmGF14,* and *OsGF14f* transgenic plants to drought stress indicates that 14-3-3 proteins play a core role under water-deficient conditions [23,24,25]. Hv14-3-3A responds to drought conditions via rapid stomatal regulation in barley [3]. OsGF14b plays a key function in responding to salt stress by interacting with OsPLC1 and promoting phospholipase C1 stability [26]. OsGF14b confers resistance to rice under drought stress, and the mechanism is partially dependent on abscisic acid (ABA) [27,28]. In transgenic tobacco, TaGF14b strengthens the resistance to drought and salt stress by increasing the ABA content and promoting ABA signaling [29].

In addition, the ABA-responsive element (ABRE)-binding factor (ABF) ABI5 interacts with three 14-3-3 proteins in *Brachypodium distachyon* (*B. distachyon*), and BdABRE/ABF belonging to bZIP transcription factors (TFs) interacts with 14-3-3 proteins, thus functioning in abiotic stress responses [30,31]. Numerous studies have shown that 14-3-3 proteins are affected by ABA, as well as affecting ABA signaling [32,33,34,35,36]. However, little evidence validating the relationship between the abiotic stress response exhibited by plant 14-3-3 proteins and the involvement of ABA is available.

Although progress has been made in elucidating the roles of plant 14-3-3 proteins, few 14-3-3 proteins have been functionally studied in *B. distachyon*, an emerging monocotyledonous plant model [37]. In this research, *BdGF14g*, a *14-3-3* gene, was identified in *B. distachyon*. *BdGF14g*-expressing tobacco exhibited enhanced tolerance to drought because of an improved reactive oxygen species (ROS)-scavenging ability, rapid stomatal closure, and regulated ABA accumulation and signaling. Moreover, we verified the connection between the *B. distachyon 14-3-3* gene and ABA signaling in coping with water deficiency.

## 2. Results

### 2.1. BdGF14g Is Located in the Cytoplasm and Nucleus

The mixture of complementary DNA (cDNA) templates from two-week-old *B. distachyon* seedlings after treatments was used to clone the sequence of a 14-3-3 gene named BdGF14g, belonging to the non-ε subgroup, with an ORF of 789 bp and encoding a 262 aa protein (BRADI5G12510.2; GenBank: KU933263.1). The ORF of *BdGF14g* was cloned into *pBI121*-*GFP* (VC) driven by the promoter of CaMV 35s, with gene-specific primers listed in Table S1.

**Figure 1:**
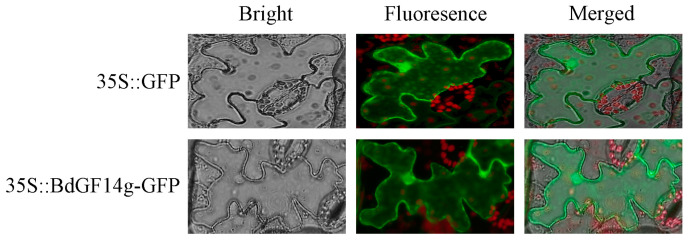
Subcellular localization of BdGF14g::GFP fusion protein and GFP in tobacco epidermal cells. The plasmids of *pBI121*-*BdGF14g*-*GFP* and *PBI121*-*GFP* were introduced into EHA105, an *Agrobacterium* strain, and then injected into tobacco cells via *Agrobacterium* transformation. After incubating for 48 h, photos were taken under an inverted fluorescence microscope.

To find the subcellular location of BdGF14g in plant cells, VC and *pBI121-BdGF14g-GFP* plasmids were transferred into tobacco (*Nicotiana tabacum* L.) from the abaxial surface of leaves via *Agrobacterium* transformation. Consistent with the control (pBI121-GFP), a green fluorescence signal of pBI121-BdGF14g-GFP was detected in tobacco cells (Figure 1). These results indicate that BdGF14g was located throughout the tobacco plant cell.

### 2.2. BdGF14g Improves Drought Tolerance at the Young Seedling Stage

**Figure 2:**
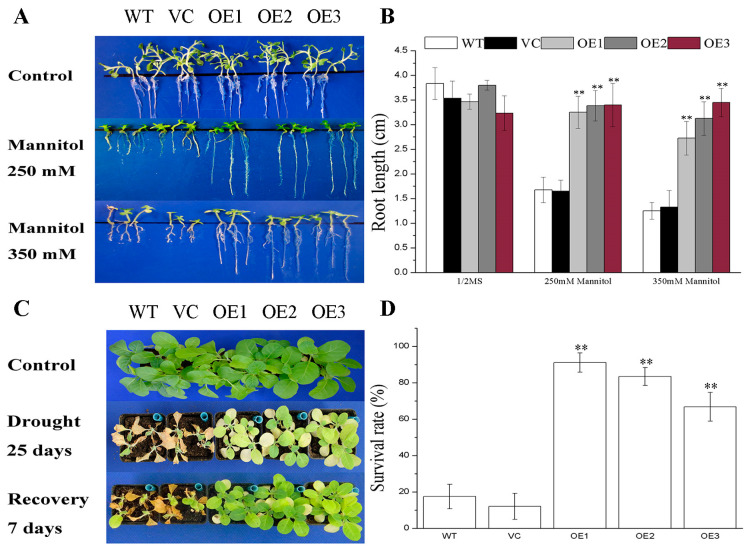
Response of *BdGF14g* transgenic tobacco plants to drought stress. (**A**) The seedlings of wild type (WT), vacant vector (VC) controls, and three *BdGF14g*-overexpressing lines after seven days of germination were planted on 1/2 MS with 250 mM or 350 mM of mannitol. After vertical cultivation for two weeks, the root lengths of at least nine seedlings per line were detected and statistically analyzed (**B**). (**C**) The two-week-old seedlings growing on MS were transplanted into pots filled with soil for three weeks under normal conditions. After that, watering was stopped for 25 days, and then they were rewatered for a further 7 days. (**D**) The respective survival rates of *BdGF14g*-expressing tobacco plants and controls suffering from water withholding were measured based on at least 35 seedlings per line in each replicate. Error bars were calculated from three independent experiments. Asterisks indicate marked differences in statistics (** *p* < 0.01).

*BdGF14g* driven by CaMV 35s was transformed into model tobacco plants to determine the role of BdGF14g in coping with drought stress. Three stable and independent T_3_ transgenic lines, detected using relative quantitative PCR (RT-qPCR), designated as OE1, OE2, and OE3 (Figure S1), were selected for stress-related studies, and then a root length assay was performed. *BdGF14g* transgenic plants and control lines had similar growth statuses, and the root lengths were not significantly different on 1/2 MS, whereas the roots of the three *BdGF14g*-expressing tobacco plants were much longer than those of the controls when grown on media containing 250 mM and 350 mM of mannitol (Figure 2A,B). These results show that *BdGF14g*-expressing tobacco plants grew better under mannitol treatment at the seedling stage.

### 2.3. BdGF14g Improves Drought Tolerance at the Mature Seedling Stage

To further confirm the responses of *BdGF14g* transgenic tobacco plants at the mature seedling stage to drought conditions, three-week-old controls and three *BdGF14g* transgenic tobacco plants growing in the soil were subjected to water-deficient conditions. After the 25 d treatment, the *BdGF14g*-expressing tobaccos exhibited better growth than the control lines, whereas the leaves of most wild-type (WT) and vacant vector control (VC) plants were wilting and yellowish (Figure 2C). After re-watering for 7 d, some transgenic plants recovered to a normal growth status, and the statistical analyses of the survival rates showed that those of the *BdGF14g*-expressing plants were remarkably higher (66–91%) than those of the controls (12–17%) (Figure 2D). These results show that *BdGF14g* transgenic tobacco exhibited increased resistance to water deficiency at the adult stage, which is in line with the results at the young seedling stage.

### 2.4. BdGF14g Increases ROS-Scavenging Ability under Water Deficiency

**Figure 3:**
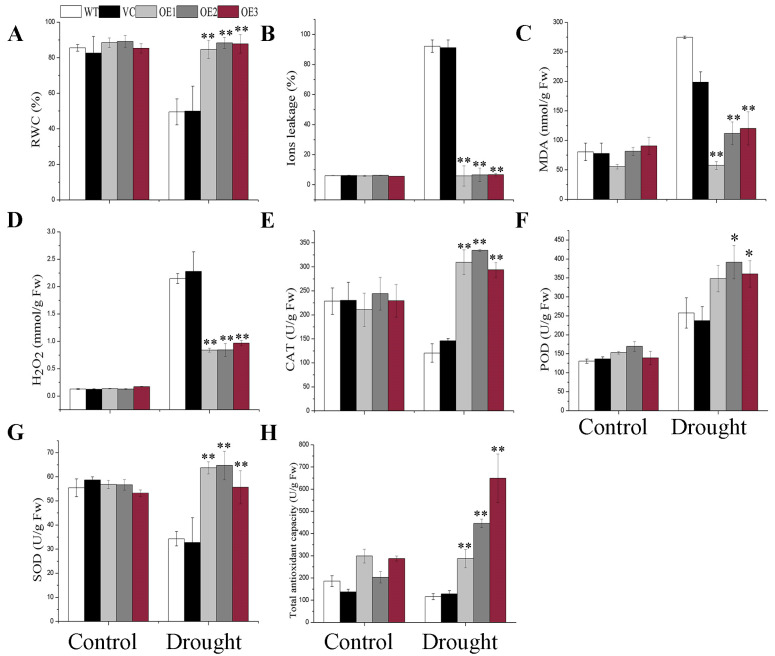
The physiological index analyses of controls and *BdGF14g* transgenic plants in response to water withholding. The two-week-old seedlings growing on MS solid medium were grown in potting soil for three weeks under normal conditions, and no water was supplied to treat the seedlings for a further fifteen days. Leaves, 0.2 g in weight, from at least three tobacco plants of test and control groups were sampled to measure the (**A**) relative water content (RWC), (**B**) malondialdehyde (MDA) content, (**C**) hydrogen peroxide (H_2_O_2_) content, (**D**) ion leakage (IL), the enzyme activities of (**E**) catalase (CAT), (**F**) peroxidase (POD), (**G**) superoxide dismutase (SOD), and (**H**) total antioxidant capacity (T-AOC). Error bars were calculated from three independent experiments. Asterisks indicate marked differences in statistics (* *p* < 0.05; ** *p* < 0.01).

The stability of the cell membrane was reduced and the ion balance was destroyed under abiotic stress. Ion leakage (IL) and malondialdehyde (MDA), two membrane damage indices, arising from ROS, as well as H_2_O_2_ content and relative water content (RWC) were measured. No pronounced differences were observed between the three *BdGF14g*-expressing plants and controls under normal conditions (Figure 3). However, compared with the controls, lower IL, MDA, and H_2_O_2_ contents, as well as higher RWCs, were observed in *BdGF14g* transgenic tobacco plants deprived of water (Figure 3A–D). These results indicate that *BdGF14g* transgenic tobacco plants suffered less cell damage caused by ROS than WT and VC plants when subjected to drought. Therefore, the enzyme activities of the antioxidant system, including catalase (CAT), peroxidase (POD), superoxide dismutase (SOD), and total antioxidant capacity (T-AOC), were measured. Under normal conditions, there was no statistical difference between any of the detected groups. When subjected to a water deficit, *BdGF14g*-expressing tobacco plants exhibited higher CAT, POD, SOD, and T-AOC activities than the controls (Figure 3E–H). The above results show that BdGF14g could mitigate oxidant-induced cell injury by increasing the activities of enzymes related to ROS scavenging, including CAT, SOD, POD, and T-AOC, under drought stress.

### 2.5. BdGF14g Overexpression Enhances Stomatal Closure under Water Deficiency and ABA Treatments

**Figure 4:**
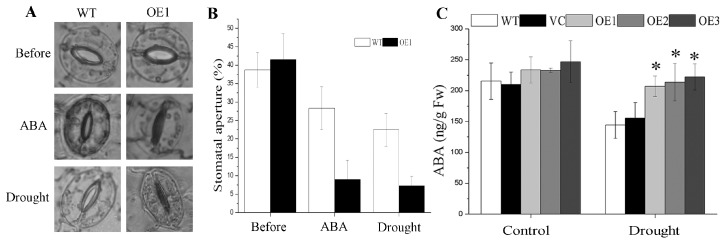
The stomatal movement of WT and *BdGF14g*-expressing line (OE1) under dehydration and ABA treatments, and abscisic acid (ABA) content under drought stress. (**A**) The stomatal movements of potted WT and OE1 lines for six weeks were detected in normal, 40 min dehydration, and 50 μM 2 h ABA conditions. Images were captured under a fluorescence microscope in a bright field. (**B**) The stomatal aperture of at least four leaves per line was examined under dehydration, ABA treatments, and control conditions. (**C**) The endogenous ABA content under normal and water-withholding conditions. The error bars were calculated based on three independent replicates. Asterisks indicate marked differences in statistics (* *p* < 0.05).

As the stomata are a major gateway for water loss via transpiration in plants, the stomatal aperture is considered another important index of tolerance to drought stress. Therefore, stomatal apertures were measured using WT and OE1 lines as examples. Before treatment, the stomata of controls and *BdGF14g*-expressing tobacco plants were open, and the stomatal apertures were similar in these lines. However, after dehydration for 40 min, the stomata of the *BdGF14g* transgenic line OE1 were almost closed, whereas those of the WT were still open, and the transgenic line showed a lower stomatal aperture (Figure 4A,B). These results indicate that *BdGF14g* transgenic tobacco plants increased the speed of stomatal closure to decrease trans-epidermal water loss and improve the tolerance to water deprivation.

ABA, a phytohormone, performs a vital function in plants coping with adverse environmental stresses [38] and functions in stomatal closure and growth regulation. To identify whether the guard cells of *BdGF14g* transgenic lines respond to ABA treatment, leaves were exposed to 50 μM of ABA for 2 h. The stomata of *BdGF14g*-expressing tobacco plants were almost closed, whereas those of most of the control plants were still open. Statistical analysis also exhibited that the controls’ stomatal apertures were larger than those of the transgenic plants when exogenous ABA was added (Figure 4A,B).

### 2.6. Drought Tolerance Conferred by BdGF14g Involving ABA in Transgenic Tobacco Plants

**Figure 5:**
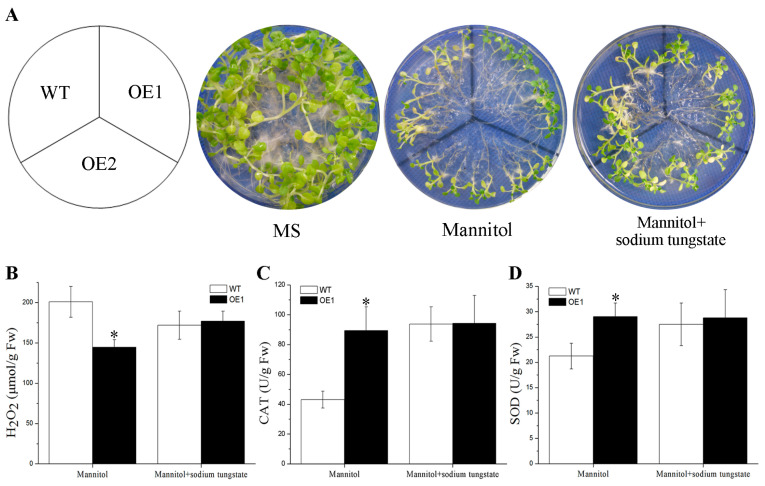
The *BdGF14g*-overexpressing lines lost the resistance to drought after adding an endogenous ABA inhibitor, sodium tungstate (Tu). Ten OE1, OE2, and WT tobacco plants of two-week-old seedlings were grown in 1/2 MS containing 350 mM of mannitol or 350 mM of mannitol + 1 mM Tu for two weeks, and then photographs were taken (**A**). Young seedlings (0.2 g, besides roots) of the transgenic line (OE1) and WT without or with Tu treatment were harvested for measurements of (**B**) H_2_O_2_, (**C**) CAT, and (**D**) SOD. Error bars were calculated from three independent experiments. Asterisks indicate marked differences in statistics (* *p* < 0.05).

To confirm the role of ABA in the response of *BdGF14g* transgenic tobacco plants to drought stress, ABA content was measured. Under normal conditions, no statistically significant difference between the controls and three transgenic plants was detected. Endogenous ABA production increased in the tested transgenic tobaccos exposed to dehydration stress compared with that in the controls (Figure 4C). In addition, sodium tungstate (Tu), an ABA biosynthesis inhibitor, was chosen to further study the phenotypes (OE1 and OE2) and physiological indices (OE1, randomly selected) of two-week-old *BdGF14g* transgenic tobacco plants in response to drought treatment. Under mannitol stress, the *BdGF14g* transgenic tobacco plants showed a better growth status, with lower H_2_O_2_ contents and higher activities of CAT and SOD than the WT plants; however, when Tu was added, the notable differences between transgenic tobacco OE1 and WT plants in growth status, enzyme activities, and H_2_O_2_ content under mannitol stress disappeared (Figure 5). The above results indicate that BdGF14g participated in ABA-regulated stomatal closure, and when inhibiting endogenous ABA synthesis in *BdGF14g*-overexpressing tobacco plants, the tolerance and higher antioxidase activities in transgenic tobacco plants decreased under drought treatment, implying that the higher resistance to drought stress conferred by BdGF14g was dependent on ABA.

### 2.7. BdGF14g Interacts with NtABF2 and Increases the Expression of ABA-Related Gene in Transgenic Tobacco Plants

**Figure 6:**
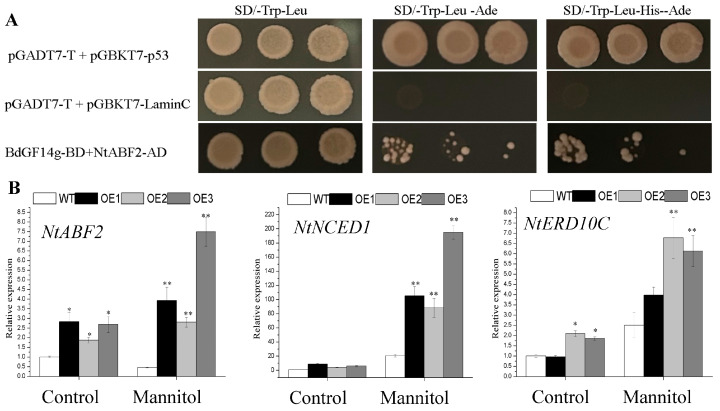
BdGF14g interacted with NtABF2 in the yeast two-hybrid assay and transcript expression analyses of the ABA signaling pathway. (**A**) BdGF14g was transferred into the pGBKT7 vector, and NtABF2 was cloned into the pGADT7 vector, followed by their co-transfection into the AH109 strain, and spotted onto the nutritional selective solid media SD/-Trp-Leu, SD/-Trp-Leu-Ade, and SD/-Trp-Leu-His-Ade. The transformants pGADT7-*T*, pGBKT7-*p53*, and pGBKT7-*LaminC* represent the controls. (**B**) The transcript expression levels of ABA-related genes *NtABF2*, *NtNCED1*, and *NtERD10C*. The error bars were calculated based on three independent replicates. Asterisks indicate marked differences in statistics (* *p* < 0.05; ** *p* < 0.01).

ABF2, an important transcriptional regulator of ABRE-dependent ABA signaling, is involved in the response to water deficit. In our previous studies, BdGF14g was shown to interact with three BdABRE/ABF TFs. Therefore, a yeast two-hybrid (Y2H) assay was performed to detect the interaction between BdGF14g and NtABF2. The results exhibit that NtABF2 interacted with BdGF14g in SD/-Trp-Leu-Ade and SD/-Trp-Leu-His-Ade solid media (Figure 6A).

To further elucidate the molecular mechanism of *BdGF14g* in response to drought conditions, the expression analyses of *NtABF2*, *NtNCED1*, a crucial ABA biosynthesis gene, as well as *NtERD10C*, an ABA-related stress defense gene, were performed. All detected genes (in addition to *NtNCED1* under control conditions) were markedly higher in *BdGF14g*-expressing tobacco plants compared with WT plants under normal and drought conditions, and their expressions were elevated with dehydration (Figure 6B). These results show that BdGF14g improved drought tolerance by interacting with NtABF2 and upregulating the expression of genes related to ABA signaling.

## 3. Discussion

To cope with many abiotic stresses, including drought, plants generate complex signal transduction networks to respond to these abiotic stresses [39,40]. The 14-3-3 family proteins participate in various physiological processes, including plant hormone and light signaling, basic metabolism, biotic stress, and so on [6,11,13,20,41,42]. In addition, an increasing number of 14-3-3 proteins have been found to play cardinal roles in plants that cope with adverse abiotic stresses. In the present study, a 14-3-3 protein, BdGF14g, was extracted from *B. distachyon* located in the whole tobacco cell (Figure 1), and it was similar not only to the reported 14-3-3 proteins in *B. distachyon,* BdGF14d and BdGF14b [24,43], but also to 14-3-3 proteins in other plants such as At14-3-3λ and OsGF14f [14,25,44]. The cytoplasm nucleus localization of 14-3-3λ-GFP had no obvious change under different light conditions [14], but significantly strong GFP signals were observed in the nuclei of root cells after 4 °C treatment [44].

*BdGF14g* can be upregulated by PEG and ABA and downregulated by H_2_O_2_, suggesting that BdGF14g possesses potential functions in response to these stresses [30,45,46]. Overexpressing BdGF14a and MdGRF13 exhibited enhanced resistance to drought stress in *Arabidopsis* [46,47]. Likewise, in transgenic tobacco, the function of BdGF14g in abiotic stress was first confirmed by the improved drought-tolerant phenotype in the young and adult seedling stages, with a better growth status, higher survival rate, higher RWC, and longer roots (Figure 2).

Drought treatment can lead to the overproduction of ROS, an important signal molecule in adaptive stress responses to dehydration, the overaccumulation of which may also damage plant cells [48]. Therefore, ROS-related physiological indices were determined herein. The results suggest that *BdGF14g*-expressing tobacco plants exhibited stronger antioxidase activities, including POD, CAT, SOD, and T-AOC, in response to drought stress (Figure 3). Previous studies have shown that GPX, APX, SOD, and CAT are involved in ROS detoxification [29]. Therefore, under drought stress, the higher antioxidase activities in *BdGF14g*-overexpressing tobacco plants showed that BdGF14g conferred drought tolerance by mediating the ROS-scavenging system. Consequently, lower accumulations of IL (an index of membrane injury), MDA (an index of ROS-mediated lipid peroxidation injury in the membrane), and H_2_O_2_ were detected, which indicated that cell membrane stability was strengthened in *BdGF14g*-overexpressing tobacco plants under drought stress than in WT and VC plants (Figure 3A–H). The above results, in line with those of MdGRF11 and MdGRF13, two 14-3-3 proteins detected in apples under abiotic stress in transgenic plants [47,49], indicate that BdGF14g enhanced antioxidant capacity to improve the ROS-scavenging system under drought conditions.

Avoiding the lowering of the water potential caused by constant exposure to drought is mainly based on the absorption maximization of water from the roots and water loss minimization from evapotranspiration from leaves [39,50]. An enhanced root length benefits the uptake of water under adverse conditions, improving plant adaptation to drought stress [50]. Furthermore, stomatal regulation is a vital step to decrease the loss of water under water shortage conditions [3,38]. In this study, in accordance with the higher RWC, longer roots and lower stomatal apertures of *BdGF14g* transgenic plants under drought stress were observed (Figure 2A, Figure 3A and Figure 4A,B). These results are partially in line with the previously reported results that At14-3-3λ, ZmGF14-6, GRF9, GsGF14o, TaGF14b, and Hv14-3-3A conferred resistance to plants to extreme stress partly via stomatal regulation and root elongation [3,22,23,24,29,51]. Hence, the above findings show that *BdGF14g* improved the water retention capacity (higher RWC) and enhanced transgenic tobacco plants’ tolerance to dehydration by accelerating stomatal closure and increasing root length.

ABA exhibits a cardinal function in plants that cope with abiotic stresses by participating in stomatal closure, growth regulation, and seed germination inhibition [36,38,52]. The 14-3-3 protein interacts with ABF TFs and participates in responding to adverse environmental conditions via the ABA signaling pathway [30,31,32,34,51]. In our previous study, *BdGF14g* was upregulated by exogenous ABA. In this study, endogenous ABA production in *BdGF14g* transgenic seedlings was higher than in controls subjected to water deficiency, and the stomata of *BdGF14g*-overexpressing tobacco plants closed faster under drought and exogenous ABA treatments (Figure 4A,B). These findings imply that BdGF14g may participate in ABA signaling under dehydration treatment. We previously reported that BdGF14g interacted with three BdABRE/ABF TFs, including BdbZIP62, an ABA-insensitive 5 (ABI5)-like protein [30]. In *Arabidopsis*, ABI5 functions in ABA signaling and ROS homeostasis during seed germination [53]. Herein, when Tu, an ABA biosynthesis inhibitor, was added, the better growth status and higher enzyme activity of transgenic tobacco plants than those of WT under mannitol treatment disappeared, and no notable differences were observed (Figure 5). These results are also in accordance with previous results showing that ABA enhances the activities of ROS-scavenging enzymes to alleviate oxidative damage [29,54]. The above results indicate that BdGF14g functions in ABA-regulated stomatal closure and suggest that BdGF14g might improve antioxidant enzyme activity in transgenic tobacco via the ABA signaling pathway to achieve tolerance to drought stress.

MdGRF11, a 14-3-3 gene in apples, interacts with MdAREB/ABF TFs, and MdGRF11 improves the drought stress resistance of the MdGRF11-overexpressing plant by altering the transcription of ABA-signaling marker genes [49]. OsGF14f positively mediates ABA responses induced by drought stress by interacting with OsbZIP23, a notable AREB/ABF TF, to enhance the transcriptional regulatory activity of downstream target genes [25,55]. In line with previous results, in this study, the expression of the ABA-related stress defense genes, *NtABF2,* a critical ABRE-dependent transcriptional regulator of ABA signaling, *NtNCED1*, a crucial ABA biosynthesis gene, and *NtERD10C,* an ABA-related stress defense gene [56,57], were obviously induced in BdGF14g-overexpressing plants under drought treatment (Figure 6B), indicating that BdGF14g positively regulated ABA signal transduction at the transcriptional level. Moreover, similar to the interactions of MdGRF11 and OsGF14f with ABRE/ABFs in response to abiotic stress [25,49], BdGF14g also interacted with NtABF2 (Figure 6A).

ABA-induced ABF2 and its homologs play master roles in the presence of drought stress by modulating stomatal aperture, root length, antioxidant activity, ABA content, downstream stress-responsive gene expression, osmotic regulator accumulation, transpiration rate, photosynthetic capacity, and germination [50,58,59,60]. This is similar to the response of BdGF14g to drought in the present study. Moreover, the AREB/ABF TFs regulate the transcription of downstream ABA-responsive genes by interacting with the ABRE elements of the promoters [25,55,58,59]. For example, PtrABF2 directly binds to the promoter of pADC containing ABRE core sequences to respond positively to drought stress [59]. OsbZIP23 directly promotes the expression of OsNCED4, a vital ABA biosynthesis gene, by binding to its promoter with ABRE elements to regulate the ABA response, and the interaction of OsbZIP23 with OsGF14f can enhance this transcriptional activity [25,55]. NtNCED1, whose promoter contains an ABRE element, is a putative NtABF2 direct target gene and might be a critical gene in the feedback regulation of ABA accumulation to enhance the ABA-dependent drought tolerance conferred by BdGF14g. The observed increase in ABA content in BdGF14g-overexpressing lines under drought stress (Figure 4C) partly proved the validity of this hypothesis, although further experiments, including yeast one-hybrid (Y1H), dual luciferase, and electrophoretic mobility shift assays, need to be performed to further confirm this hypothesis.

## 4. Materials and Methods

### 4.1. Plant Materials and Stress Treatments

*Nicotiana tabacum* L. (Samsun) was used for genetic transformation and stress tolerance analyses. The T_3_ seeds of *BdGF14g* transgenic tobacco, VC, and WT were sterilized according to previously reported instructions [29]. The sterilized seeds were germinated on 1/2 MS without vitamins in Petri dishes. The dishes were then kept in a growth chamber (16 h light/8 h dark, 120 µmol photons m^−2^ s^−1^, and 22 °C). The two-week-old tobacco plants were then potted in vermiculite in a phytotron in 16 h light/8 h dark and 150 µmol photons m^−2^ s^−1^ at 25 °C.

### 4.2. Cloning and Subcellular Localization Analyses of BdGF14g

*BdGF14g* was cloned with the cDNA template reverse transcribed from a mixture of RNA extracted from *B. distachyon* seedlings after ABA, H_2_O_2_, NaCl, and PEG6000 treatments. The ORF of *BdGF14g* was cloned into *pBI121*-*GFP* (VC) driven by the promoter of CaMV 35s. The *pBI121*-*BdGF14g-GFP* and VC plasmids were separately transformed into EHA105 (an *Agrobacterium tumefaciens* strain) and injected into tobacco plants from the lower epidermal cells according to a previously described method [61]. Images were obtained after incubation in a growth chamber for 48 h. The gene-specific primers used are listed in Supplementary Table S1.

### 4.3. Tobacco Transformation and Stress Tolerance Analyses

The *pBI121*-*BdGF14g-GFP* and VC plasmids were separately transformed into EHA105 and then transformed into tobacco plants via the reported *Agrobacterium*-mediated transformation [62]. After using a medium containing 100 mg/L of kanamycin (Kan) for screening, the expression levels in *BdGF14g* transgenic lines were assayed with RT-qPCR, with *NtGAPDH* as the control. The T_3_ seeds of three stable independent *BdGF14g* transgenic tobacco plants (OE1, OE2, and OE3) were harvested and sprouted on the 1/2 MS conventional medium (containing 100 mg/L of Kan) for abiotic-stress-tolerance-related assays. The one-week-old seedlings of *BdGF14g*-expressing lines and controls were transferred to 1/2 MS and 1/2 MS with 250 mM or 350 mM of mannitol, respectively, and the root lengths of at least nine seedlings per line were measured after vertical cultivation for two weeks. For the drought resistance assay during early seedling development, the seedlings were transferred to 1/2 MS, 1/2 MS + 350 mM mannitol, and 1/2 MS + 350 mM mannitol + 1 mM Tu for two weeks, and 0.2 g of plantlets (besides the roots) was harvested for further physiological analysis. For the drought treatment, two-week-old geminated tobacco plants grown in pots (five seedlings/pot) filled with vermiculite for 3 weeks were exposed to dehydration for 25 d and then re-watered for 7 d to take photos, and 0.2 g of leaves from at least three seedlings per line without water supplied for 15 d was sampled for further physiological measurements.

### 4.4. Measurements of Aridity-Responsive Physiological Parameters

To investigate the differences in physiological indices of *BdGF14g*-expressing tobacco plants in adaptation to dehydration, 0.2 g of leaves or seedlings was collected and ground in 1.8 mL of phosphate-buffered saline on ice. The supernatant was obtained via centrifugation (12,000 rpm for 20 min). The contents of H_2_O_2_ and MDA and antioxidase activities of T-AOC, POD, CAT, and SOD, were measured with the respective assay kits (Comin, Suzhou, China). The ABA content was assessed using the ELISA kit (Jiancheng, Nanjing, China). The IL and RWC were also calculated in terms of a priorly reported method [54]. The formula IL (%) = C1/C2 × 100 was used to calculate the IL.

### 4.5. Stomatal Closure Assay

A stomatal closure assay was performed according to a prior report [63]. The leaves of four-week-old tobacco plants (WT, VC, and OE) were immersed in a solution containing 50 μM of CaCl_2_, 10 mM of MES-KOH, and 30 mM of KCl (PH 6.15) and put under high-intensity light (200 μmol m^−2^ s^−1^) for 6 h to make the stomata open, and then the leaves were removed to dehydrate at 25 °C for 40 min or put into a solution containing 50 μM of ABA for 2 h. After treatments, the stomatal statuses were observed under a fluorescence microscope (IX71, Olympus, Tokyo, Japan) [29]. The width/length rates of at least four leaves per line were calculated in each biological replicate to indicate the stomatal aperture.

### 4.6. Y2H Assays

The Y2H assay was performed to detect the interaction between BdGF14g and NtABF2 according to previously reported methods [64]. Full-length coding sequences of *BdGF14g* were cloned into the pGBKT7 (BD) vector, and *NtABF2* was transferred to the pGADT7 (AD) vector. Next, these vectors were used to co-transform AH109 (a yeast strain), plated on the SD/-Trp-Leu selection medium for 3 d, spotted on the nutritional selective solid media SD/-Trp-Leu, SD/-Trp-Leu-Ade, and SD/-Trp-Leu-His-Ade, and grown at 30 °C for 3–5 d. The *pGBKT7-p53* or *pGADT7-T,* and *pGBKT7-LaminC* transformants were taken as the negative and positive control, respectively. The interaction was estimated by the growth ability of yeast cells on selective plates of SD/-Trp-Leu-Ade and SD/-Trp-Leu-His-Ade. Detailed information on the related gene-specific primers is presented in Table S1.

### 4.7. qRT-PCR Assays

To examine the expressions of ABA-related stress-responsive genes in *BdGF14g*-expressing lines and WT, three-week-old seedlings were transferred to a medium of 1/2MS + 300 mM mannitol for 1 week. These stress-related genes included the ABA-related genes *NtABF2*, *NtNCED1*, and *NtERD10C*. RNA was extracted using an RNA extraction kit (Zoman Biotechnology, Beijing, China), and cDNA was synthesized using a FastQuant RT kit (Tiangen, Beijing, China) [29]. Detailed information on the gene-specific primer pairs is provided in Table S2 [29]. For data analyses, the 2^−ΔΔCT^ method was used to compute the relative expressions [65].

### 4.8. Statistical Analyses

GraphPad Prism (GraphPad Software 6.01, Inc., La Jolla, CA, USA) was used for statistical analyses following the reported methods [29].

## 5. Conclusions

**Figure 7:**
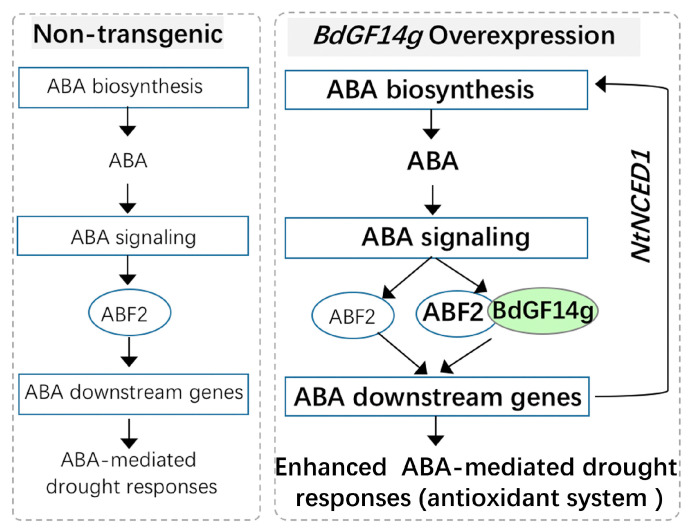
A proposed mechanism of BdGF14g involved in regulating ABA-signaling-mediated drought tolerance. BdGF14g interacts with NtABF2, the transcription and activation of which can be enhanced by drought-induced ABA accumulation mediated by ABA-signaling genes, including an ABA-synthesis-related gene, NtNECD1, resulting in increased ABA production, which, in turn, regulates the ABA signaling pathway via a feedback mechanism. BdGF14g interacts with NtABF2 and is involved in upregulating ABA-related genes to enhance drought resistance.

In conclusion, BdGF14g is an adaptor protein that enhances drought resistance in transgenic tobacco via increasing membrane stability, modulating the antioxidase system activities, improving the water retention capacity by regulating ABA signaling and accumulation, and increasing the expression of ABA-related stress genes, including *NtNCED1*, a critical ABA biosynthesis gene. Based on these results, a probable mechanism explaining the role of BdGF14g in the ABA response to drought stress was proposed (Figure 7). ABA accumulation induced by water deficit increases the transcription and activation of NtABF2. BdGF14g interacts with NtABF2 and then regulates the downstream genes, including NECD1, promoting ABA accumulation and generating feedback regulation to enhance ABA-signaling-dependent drought tolerance. These results show that drought stress adaptation conferred by BdGF14g is closely related to its interaction with NtABF2 and the modulation of ABA-signaling-related genes. We speculate that BdGF14g may be involved in the ABA signaling pathway, partly via its interaction with NtABF2 and the regulation of ABA-signaling-related genes.

## 6. Patents

Guangyuan He, Guangxiao Yang, Junli Chang, Yuan He, Yang, Zhang, Huazhong University of Science and Technology. A drought resistance gene and expression vector of *Brachypodium distachyon* and the encoded protein and application: CN, [P]. ZL 201711426688. X, 2020.12.18.

## Data Availability

Data are contained within the article.

## Supplementary Materials

The following supporting information can be downloaded at https://www.mdpi.com/article/10.3390/plants12233975/s1. Table S1: Primers for constructions of pBI121-*BdGF14g*, pGBKT7-*BdGF14g,* and pGADT7-*NtABF2*; Table S2: Primers for expressions of related marker genes; Figure S1: The relative expression levels of *BdGF14g* in transgenic tobacco plants were detected.
