# Supplementary material for: A 14-3-3 Protein-Encoding Gene, BdGF14g, Confers Better Drought Tolerance by Regulating ABA Biosynthesis and Signaling
Source: Plants (Basel). 2023 Nov 26;12(23):3975. doi: 10.3390/plants12233975 (PMC10707786; doi:10.3390/plants12233975)
Supplement: Supplementary file 1 [file plants-12-03975-s001.zip › plants-2705875-supplementary.pdf]

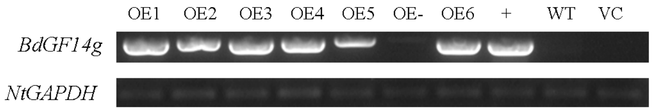

Figure S1. The relative expression levels of *BdGF14g* in transgenic tobaccos were detected. The *NtGAPDH* was taken as the internal reference.

Table S1. Primers for constructions of pBI121-*BdGF14g,* pGBKT7-*BdGF14g* and pGADT7-*NtABF2*

Gene Name	Forward/ Reverse primers(5'-3')
pBI121-*BdGF14g*	GCTCTAGAATGTCGGCACCTGCGGAGCT
	CGGGATCCCTGCCCATCACCAGAGTCA
BdGF14g-BD	GGATCCCG ATGTCGGCACCTGCGGAGCT
	AACTGCAGCTGCCCATCACCAGAGTCA
NtABF2-AD	CCATGGAGGCCAGTGAATTCATGGGGAGTAATTTTAATT
	CAGCTCGAGCTCGATGGATCCTTACCATGGACCAGTCTG

Table S2. Primers for expressions of related marker genes

Gene Name	Forward/ Reverse primers(5'-3')
NtGAPDH	TGCCTTGAGCAAGAACTTTGTG
	GGCAGATCAAATCAATCACACG
NtERD10C	AACGTGGAGGCTACAGATCG
	GTTCCTCTTGGGCATGAGTT
NtNCED1	AAGAATGGCTCCGCAAGTTA
	GCCTAGCAATTCCAGAGTGG
NtABF2	GCAGCCATCTATCTATTC
	GCAACTCATCCATATTCA
